# Integrating Sequencing Technologies in Personal Genomics: Optimal Low Cost Reconstruction of Structural Variants

**DOI:** 10.1371/journal.pcbi.1000432

**Published:** 2009-07-10

**Authors:** Jiang Du, Robert D. Bjornson, Zhengdong D. Zhang, Yong Kong, Michael Snyder, Mark B. Gerstein

**Affiliations:** 1 Department of Computer Science, Yale University, New Haven, Connecticut, United States of America; 2 Keck Biotechnology Resource Laboratory, Yale University, New Haven, Connecticut, United States of America; 3 Department of Molecular Biophysics and Biochemistry, Yale University, New Haven, Connecticut, United States of America; 4 Department of Molecular, Cellular and Developmental Biology, Yale University, New Haven, Connecticut, United States of America; 5 Program in Computational Biology and Bioinformatics, Yale University, New Haven, Connecticut, United States of America; University of Washington, United States of America

## Abstract

The goal of human genome re-sequencing is obtaining an accurate assembly of an individual's genome. Recently, there has been great excitement in the development of many technologies for this (e.g. medium and short read sequencing from companies such as 454 and SOLiD, and high-density oligo-arrays from Affymetrix and NimbelGen), with even more expected to appear. The costs and sensitivities of these technologies differ considerably from each other. As an important goal of personal genomics is to reduce the cost of re-sequencing to an affordable point, it is worthwhile to consider optimally integrating technologies. Here, we build a simulation toolbox that will help us optimally combine different technologies for genome re-sequencing, especially in reconstructing large structural variants (SVs). SV reconstruction is considered the most challenging step in human genome re-sequencing. (It is sometimes even harder than de novo assembly of small genomes because of the duplications and repetitive sequences in the human genome.) To this end, we formulate canonical problems that are representative of issues in reconstruction and are of small enough scale to be computationally tractable and simulatable. Using semi-realistic simulations, we show how we can combine different technologies to optimally solve the assembly at low cost. With mapability maps, our simulations efficiently handle the inhomogeneous repeat-containing structure of the human genome and the computational complexity of practical assembly algorithms. They quantitatively show how combining different read lengths is more cost-effective than using one length, how an optimal mixed sequencing strategy for reconstructing large novel SVs usually also gives accurate detection of SNPs/indels, how paired-end reads can improve reconstruction efficiency, and how adding in arrays is more efficient than just sequencing for disentangling some complex SVs. Our strategy should facilitate the sequencing of human genomes at maximum accuracy and low cost.

## Author Summary

In recent years, the development of high throughput sequencing and array technologies has enabled the accurate re-sequencing of individual genomes, especially in identifying and reconstructing the variants in an individual's genome compared to a “reference”. The costs and sensitivities of these technologies differ considerably from each other, and even more technologies are expected to appear in the near future. To both reduce the total cost of re-sequencing to an affordable point and be adaptive to these constantly evolving bio-technologies, we propose to build a computationally efficient simulation framework that can help us optimize the combination of different technologies to perform low cost comparative genome re-sequencing, especially in reconstructing large structural variants, which is considered in many respects the most challenging step in genome re-sequencing. Our simulation results quantitatively show how much improvement one can gain in reconstructing large structural variants by integrating different technologies in optimal ways. We envision that in the future, more experimental technologies will be incorporated into this simulation framework and its results can provide informative guidelines for the actual experimental design to achieve optimal genome re-sequencing output at low costs.

## Introduction

The human genome is comprised of approximately 6 billion nucleotides on two pairs of 23 chromosomes. Variations between individuals are comprised of ∼6 million single nucleotide polymorphisms (SNPs) and ∼1000 relatively large structural variants (SVs) of ∼3 kb or larger and many more smaller SVs are responsible for the phenotypic variation among individuals [1],[2]. Most of these large SVs are due to genomic rearrangements (e.g. duplication and deletion), and a few others contain novel sequences that are not present in the reference genome [3]. The goal of personal genomics is to determine all these genetic differences between individuals and to understand how these contribute to phenotypic differences in individuals.

Making personal genomics almost a reality over the past decade, the development of high throughput sequencing technologies has enabled the sequencing of individual genomes [3],[4]. In 2007, Levy et al. reported the sequencing of an individual's genome based on Sanger [5] whole-genome shotgun sequencing, followed by *de novo* assembly strategies. Wheeler et al. in 2008 presented another individual's genome sequence constructed from 454 sequencing reads [6] and comparative genome assembly methods. In the mean time, other new sequencing technologies such as Solexa/Illumina sequencing [7] have become available for individual genome sequencing with corresponding, specially-designed sequence assembly algorithm designed [8]–[12].

These projects and algorithms, however, mostly relied on a single sequencing technology to perform individual re-sequencing and thus did not take full advantage of all the existing experimental technologies. Table 1 gives a summary of the characteristics of several technologies in comparative individual genome sequencing. At one extreme, performing long Sanger sequencing with a very deep coverage will lead to excellent results at high cost. In another, performing only the inexpensive and short Illumina sequencing may generate good and cost-efficient results in SNP detection, but will not be able to either unambiguously locate some of the SVs in repetitive genomic regions or fully reconstruct many of the large SVs. Moreover, array technologies such as the SNP array [1] and the CGH array at different resolutions [13]–[16] can also be utilized to identify the SVs: the SNP arrays can detect SNPs directly, and the CGH array is able to detect kilobase-(kb) to megabase-(mb) sized copy number variants (CNV) [17], which can be integrated into the sequencing-based SV analysis. It is thus advantageous to consider optimally combining all these experimental techniques into the individual genome re-sequencing framework and to design experiment protocols and computational algorithms accordingly.

**Table 1:** Characteristics of different sequencing/array technologies in comparative individual genome sequencing.

		Long Sequencing	Medium Sequencing	Short Sequencing	CGH array (high/low resolution)
**Read length (bases)**		∼800	∼250	∼30	Tiling step size: ∼85 bp
**Approximate cost per base ($)**		∼1E-3	∼7E-5	∼7E-6	∼3E-7 per array
**Error rate per base**		0.001–0.002%	0.3–0.5%	0.2–0.6%	N/A (detecting signals rather than sequences)
**Major error type**		Substitution errors	Insertion/deletion errors (usually caused by homo-polymers)	All error types	Array-specific errors (cross-hybridization effects)
**Characteristics in comparative individual genome sequencing**	**Single reads**	Identify small / medium SVs; localize SVs close to highly represented genomic regions	Identify small SVs; localize SVs in highly represented ∼100mers	Identify SNPs; localize SNPs in lowly represented genomic regions	Detect large CNVs with relatively low resolution; relatively cheaper than current sequencing technologies
	**Paired-end reads**	Detect large Indels with relatively low resolution; provide extra information to localize SVs	Detect large Indels with relatively low resolution; provide extra information to localize SVs	Link distant SNPs for haplotype phasing	

Data based on:

1) de la Vega FM, Marth GT, Sutton GG (2008) ‘Computational Tools for Next-Generation Sequencing Applications’, Pacific Symposium on Biocomputing 2008.

2) de Bruin D (2007) UBS Investment Research, Q Series: DNA Sequencing, UBS, New York, 2007.

Due to the existence of reference genome assemblies [18],[19] and the high similarity between an individual's genome and the reference [3], the identification of small SVs is relatively straightforward in comparative re-sequencing with the analysis of single split-reads covering small SVs. Meanwhile, although there exist algorithms to detect large SVs with paired-end reads [2], the complete reconstruction of a large SV requires the integration of reads spanning a wide region, often involving misleading reads from other locations of the genome. If there were no repeats or duplications in the human genome, the reconstruction of such large SVs would be trivially accomplished by the *de novo* assembly with a high coverage of inexpensive short reads around these regions. With the existence of repeats and duplications in the human genome, however, a set of longer reads will be required to accurately locate some of these SVs in repetitive regions, and a hybrid re-sequencing strategy with both comparative and *de novo* approaches will be necessary to identify genomic rearrangement events such as deletions and translocations, and also to reconstruct large novel insertions in individuals. Such steps are thus much harder than the others, and will be the main focus of this paper.

Here we present a toolbox and some representative case studies on how to optimally combine the different experimental technologies in the individual genome re-sequencing project, especially in reconstructing large SVs, so as to achieve accurate and economical sequencing. An “optimal” experimental design should be an intelligent combination of the long, medium, and short sequencing technologies and also some array technologies such as CGH. Some of the previous genome sequencing projects [20],[21] have already incorporated such hybrid approaches using both long and medium reads, although the general problem of optimal experimental design has not yet been systematically studied. While it is obvious that combining technologies is advantageous, we want to quantitatively show the potential savings based on different integration strategies. Also, since the technologies are constantly developing, it will be useful to have a general and flexible approach to predict the outcome of integrating different technologies, including the new ones coming in the future.

In the following sections, we will first briefly describe a schematic comparative genome re-sequencing framework, focusing on the intrinsically most challenging steps of reconstructing large SVs, and then use a set of semi-realistic simulations of these representative steps to optimize the integrated experimental design. Since full simulations are computationally intractable for such steps in the large parameter space of combinations of different technologies, the simulations are carried out in a framework that can combine the real genomic data with analytical approximations of the sequencing and assembly process. Also, this simulation framework is capable of incorporating new technologies as well as adjusting the parameters for existing ones, and can provide informative guidelines to optimal re-sequencing strategies as the characteristics and cost-structures of such technologies evolve, when combining them becomes a more important concern. The simulation framework is downloadable as a general toolbox to guide optimal re-sequencing as technology constantly advances.

## Results

We first briefly describe in the following subsection a systematic genome assembly strategy for the different types of sequencing reads and array signals, which is an integration of different sequence assembly and tiling array data analysis algorithms. With the most difficult steps in the assembly strategy, i.e. the reconstructions of large SVs, discussed in detail and the performance metric for such large SV reconstruction defined, we then present a semi-realistic sequencing simulation framework, which can guide the optimal experimental design, and show the results of simulations in the reconstruction of two types of large SVs.

### Schematic genome assembly strategy

The hybrid genome assembly strategy incorporates both comparative [22] and de novo methods. On one hand, most of the assembly can be done against the reference, and it will be unnecessary to perform a computationally intensive whole genome de novo assembly. Comparative approaches will be capable of identifying small SVs and large rearrangement events. On the other hand, *de novo* assembly will sometimes still be useful in reconstructing regions with large and novel SVs.

**Figure 1:**
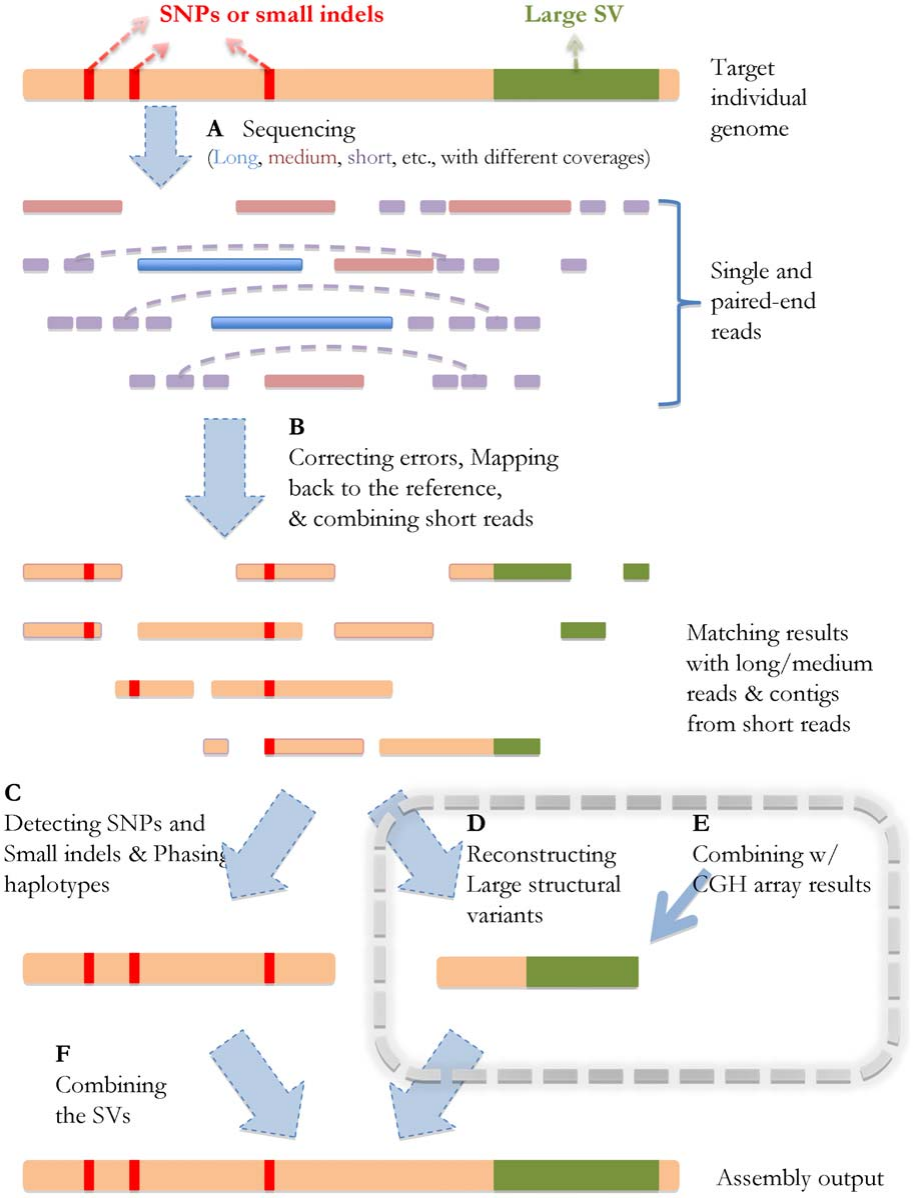
Schematic strategy of genome sequencing/assembly. The orange line represents the target individual genome, the red bars stand for the SNPs and small SVs compared to the reference, and the green region represents a large SV. (A) After the sequencing experiments, single and paired-end reads with different lengths (long, medium, short, shown in different colors) are generated, which can be viewed as various partial observations of the target genome sequence. The dashed lines represent the links of the paired-ends. The horizontal positions of the reads indicate their locations in the genome. (B) After error correction, the reads are mapped back to the reference genome, and the short reads are assembled into longer contigs based on their overlapping information. The red and green regions stand for the mismatches/gaps in the mapping results. (C) The SNPs and small SVs can be inferred directly from the mapping results, and haplotype phasing can also be performed after this step. (D, E) Large SVs can be detected and reconstructed based on the reads without consistent matches in the reference genome, and also based on the results from CGH arrays. This step will be explained in more details in the Results section. (F) The final assembly is generated after all the small and large SVs are identified.

Fig. 1 shows the schematic steps of SV reconstruction in the context of the genome sequencing/assembly process. The data from different sequencing/array experiments can be processed in the following way: As shown in Fig. 1A and 1B, with errors corrected [23] and short reads combined into “unipaths” [10], all the reads (long/medium/short) from the individual's genome can be mapped back to the reference genome. In Fig. 1C, the SNPs can then be identified immediately based on the reads with single best matches, and the boundaries of deletions or small insertions will be detected by such reads as well (allowing gaps in alignment). Meanwhile, haplotype islands can also be extracted based on the paired-end information [3],[24],[25] and the prior knowledge of the population haplotype patterns revealed by previous work [26].

**Figure 2:**
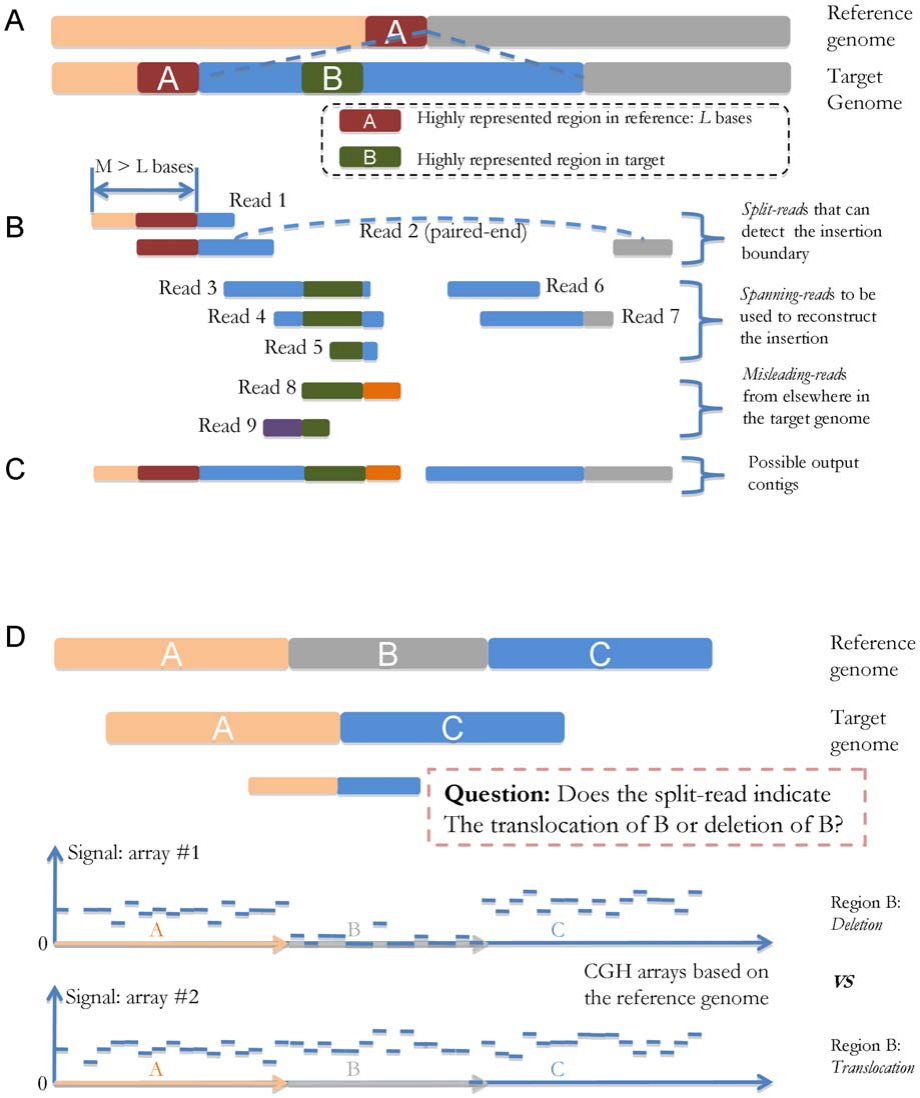
Schematic of the reconstruction of a novel insertion and rearrangement analysis. The horizontal positions of the reads indicate the mapping locations, and the colors refer to sequences from different genomic regions. (A–C) An example of the reconstruction of a novel insertion. (A) The region A (*L* bases) has multiple copies in the reference genome, and the region B has multiple copies in the target genome. The novel sequence is inserted right after a copy of region A and contains a copy of region B. (B) Split-reads such as read 1 or 2 will be needed to detect the left boundary of the insertion: read 1 is a single read that covers that boundary with *M* bases on the left (*M*>*L*); read 2 is a paired-end read with one end covering that boundary, and the two ends of read 2 can unambiguously map it back to the reference, thus revealing the insertion boundary; spanning-reads 3–7 are the reads from the novel insertion region; misleading-reads 8–9 are the reads from elsewhere in the target genome containing the same sequence contents of region B. Such reads may mislead the de novo assembly process for the novel insertion. (C) A possible set of resulting contigs after the reconstruction process. The gap is due to the false extension of the first contig caused by the misleading read 8. (D) An example of rearrangement analysis. The target individual genome has a deletion of region B from the reference. Although the sequence reads can detect such a variant, they may not be sufficient to determine whether this is a large deletion or translocation when the sequencing coverage is relatively low. The copy numbers of the genomic regions inferred from CGH array data can be integrated in the rearrangement analysis providing additional evidence of the SV types. For example, the 0 copy number of B inferred from CGH data #1 would be sufficient for us to confidently identify the deletion of B, while CGH data #2 indicates the translocation of B.

Further analysis of the single/paired-end reads are required to reconstruct the large SVs (Fig. 1D and 1E), which are by nature more complicated than identifying small SVs. First of all, locations of such SV events need to be detected by analyzing the split-reads (shown in Fig. 2A and 2B) that cover their boundaries. Second, two distinct types of SVs need to be handled separately: *de novo* assembly is required to reconstruct large novel insertions, and comparative algorithms should be utilized to identify genomic rearrangement events (e.g. segmental duplication/deletion). The homozygosity/heterozygosity of such SVs can be determined based on the existence of the reads that map back to the corresponding reference sequences.

Fig. 2A–C show the overall process of *de novo* assembly for *large novel insertions*. While the reconstruction of such regions mostly depends on the spanning-reads from the new inserted sequence, misleading-reads from elsewhere in the genome can often hinder the full reconstruction process. These reads usually comes from the highly represented regions in the genome, which also exist in the insertion. In such cases, reads longer than such regions and appropriate assembly strategies are needed to ensure the unambiguous and correct assembly output. Paired-end reads with an appropriate gap size can also help the unambiguous mapping of the reads inside novel insertions [2].

Fig. 2D illustrates the comparative identification of *rearrangements* from the reference sequence. CGH array data can be integrated into the reconstruction process of such SVs. For long rearrangements detected by sequencing data, the CGH data can be utilized in both validation and correction of large segmental duplications/deletions. What is more, incorporating the CGH data can also lower the coverage depth requirement of sequencing experiments, since the inner (i.e. not close to SV boundaries) regions of segmental duplications/deletions not covered by sequencing reads can still be identified by CNV results. An example is shown in Fig. 2D: Although the sequence reads can detect the SV event in region A, B and C, they may not be sufficient to distinguish deletions from translocations when the sequencing coverage is relatively low. The copy numbers of the genomic regions inferred from CGH array data can be integrated into the rearrangement analysis, and provide additional evidence of the SV types.

### Defining a performance metric for large SV reconstructions

It is important for us to define a reasonable performance metric so that the re-sequencing approach can be designed in such a way that its outcome will be optimized according to that metric. For large SVs, the metric can be defined based on the alignment result of the actual variant sequence and the inferred variant sequence. For a large SV due to genomic rearrangements (e.g. deletion, duplication), it is natural to define its recovery rate as either 1 (detected) or 0 (missed). For a large novel insertion, on the other hand, we may want to take into account cases where the insertion is detected but its sequence content is not reconstructed with full accuracy. Hence, we define the recovery rate of such a large novel insertion as follows based on its reconstruction percentage:
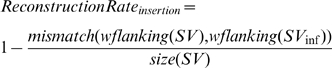
in which *SV* is the actual insertion (in simulations, it is already known; in reality, it will need to be identified in a validation step), *SVinf* is the insertion sequence inferred by the genome re-sequencing approach, *mismatch* returns the number of mismatches of two aligned sequences, *wflanking* returns a sequence with its flanking sequences on both ends, and *size* returns the size of a sequence. The purpose of introducing flanking sequences is to take into account the accuracy of the predicted location of the SV.

### Simulations of genome re-sequencing for optimal experimental design

Based on the schematic assembly strategy and the performance measure defined in the previous sections, we can simulate the sequence assembly process in order to obtain an optimal set of parameters for the design of the sequencing experiments (e.g. the amount of long (Sanger), medium (454) and short (Illumina) reads, the amount of single and paired-end reads) and the array experiments (e.g. the incorporation of CGH arrays) to achieve the desired performance with a relatively low cost in the individual genome re-sequencing project.

Here we present the results of a set of simulation case studies on reconstructing large SVs, which are in general much more challenging problems compared to the detection of small SVs. In order to fully reconstruct a long novel insertion, for instance, one needs to not only detect the insertion boundaries based on the split-reads, but also assemble the insertion sequence from the spanning- and misleading-reads. For the identification of genomic rearrangements such as deletion/translocations, one may also want to incorporate array data to increase the confidence level of such analysis. The simulations described in this section are based on large (∼10 kb, ∼5 Kb and ∼2 Kb) novel insertions and deletions discovered by Levy et al. [3], and they perform semi-realistic whole genome assembly representative using the sequence characteristics of both the NCBI reference genome [18] and the target HuRef genome [3]. The sequencing/array technologies considered in these simulations are long, medium and short sequencing methods and CGH arrays, as shown in Table 1. Paired-end reads are also included in these simulations.

One major challenge in implementing these simulations is to design them in a computationally realistic way. Brute-force full simulations of whole-genome assembly in this case would be unrealistic: thousands of possible combinations of different technologies will need to be tested, and for each of these combinations hundreds of genome assembly simulations need to be carried out to obtain the statistical distributions of their performance. Since a full simulation of one round of whole-genome assembly will probably take hundreds of CPU hours to finish, the full simulation to explore the full space of technology combinations will then require hundreds of millions (∼10^8^) of CPU hours, equivalent to ∼10 years with 1000 CPUs. We designed the simulations using analytical approximations of the whole-genome assembly process in order for them to be both time and space efficient, and the gain in efficiency is summarized in Table 2 and will be described in details later in the Materials and Methods section. We have also made this simulation framework publicly available as a toolbox that can incorporate technology advancements as well as other SV regions.

**Table 2:** Time and space complexity of different simulation strategies on the reconstruction of a large novel insertion.

Variable	Description	Representative value
G	Size of the genome	3E9 bp
c	Sequencing coverage	10×
I	Size of the large novel insertion of interest	1E4 bp
r	Average read length	50 bp
m	Average mapability values of the sub-sequences in the novel insertion	3

Simulation strategy	Number of reads generated for the reconstruction of a novel insertion	Time to compute read overlaps
Whole genome sequencing+hybrid (comparative+de novo) assembly	*O*(G×c/r) (Need to first generate all the reads from the whole genome and then perform selection)	*O*((I×c×m)^2^) (can be improved by hashing the k-mers in the reads)
Simulation utilizing pre-computed mapability maps	*O*(I×c×m/r) (simulating the reads based on the insertion region and the mapability maps)	*O*(I×c×m/r) (loss of accuracy due to the simulated misleading reads)
Approximate reduction in complexity (fold)	∼1E5	∼1.5E7

#### Case study: Large novel insertion reconstruction with shotgun reads of different lengths

**Figure 3:**
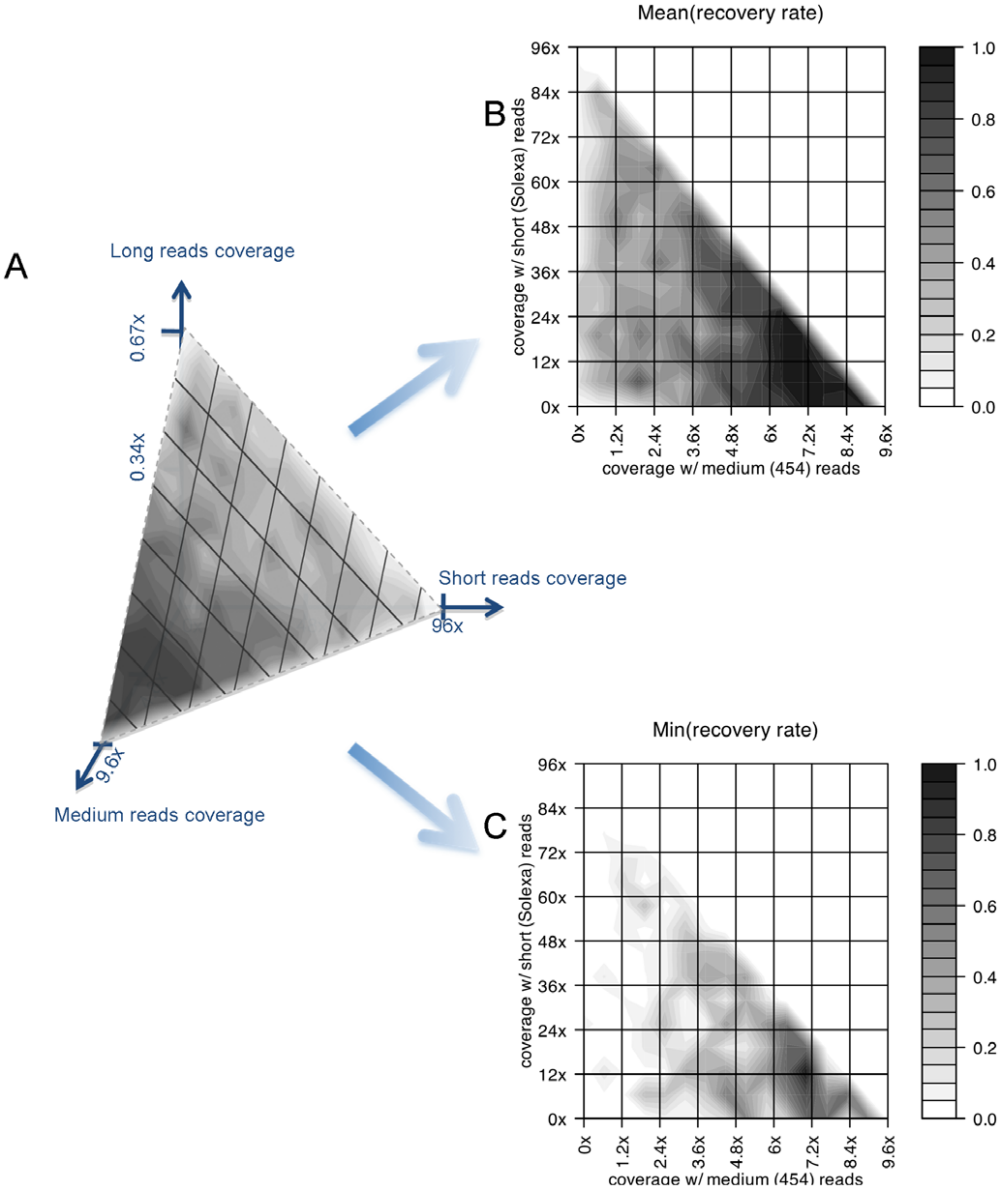
Simulation results on the reconstruction of a large novel insertion. The simulation results of the recovery rates of novel insertions when we combine long, medium and short sequencing technologies with a fixed total cost and reconstruct a ∼10 Kb novel insertion region previously identified in the HuRef genome compared to the NCBI reference genome. The total cost is ∼$7 on this novel insertion (i.e. the reads covering this region cost ∼$7), and the total re-sequencing budget is ∼$2.1 M if we scale the cost on this region to the whole genome with the same sequencing depth. (A) The triangle plane corresponds to all the sequencing combinations whose total costs are fixed. The colors on the plane indicate the average recovery rates of the novel insertion with different sequencing combinations, averaged over multiple trials of simulations. (B) The same triangle region as in Fig. 3A, projected to the 2D space with two axes representing the coverage of medium and short reads. The coverage of long reads is not explicitly shown and changes with the values of the two other two, forming a same fixed total cost as in Fig. 3A. (C) The same type of figure as Fig. 3A, showing the worst-case recovery rates on the insertion region with a fixed total sequencing cost.

Fig. 3 show the simulation results of the reconstruction of a large (∼10 Kb) novel insertion in the target individual's genome. Bear in mind that the numbers obtained are dependent on specific parameter settings of the sequencing technologies, which are summarized in Table 1. Since these technologies are evolving very rapidly (with new advancements coming out every month), these settings do not represent the current state of the art in these technologies, but are sufficient for the purpose to illustrate how our simulation approach can be used in experiment design and in combining technologies. Also, we are focusing on the full reconstruction of large novel insertions, which would in general require a higher sequencing coverage, thus a higher cost than the detection of small SVs or discovering SNPs. In these figures, the performance measures are obtained by using different combinations of long, medium and short single sequencings reads with a total cost of ∼$7 on this novel insertion (i.e. the reads covering this region cost ∼$7). The total re-sequencing budget is ∼$2.1 M if we scale the cost on this region to the whole genome with the same sequencing depth. Please note again that this $2.1 M is for illustrative purposes and does not represent the practical current “street price”. The results show that the actual performance, both average and worst-case, is heavily dependent on the coverage combination of the different technologies. The optimal performance (both average and worst-case) of sequencing/assembly is achievable when the long reads have ∼0.05× coverage, medium reads have ∼7× coverage, and short reads have ∼12× coverage (as Fig. 3C shows, the worst-case performance will decrease, i.e. the color becomes lighter, around the optimal point). A different set of simulations (results not shown) with a total budget of ∼$600 K indicate that the full reconstruction of this SV is still achievable in the optimal configuration, with an average reconstruction rate of ∼0.61.

Our simulation here is focusing on the reconstruction of large novel SVs, and thus depending on the actual characteristics of different sequencing technologies, the optimal combination of these technologies obtained in this simulation may have a trade-off in the accuracy of detecting SNPs and small indels, i.e., the optimal mixed sequencing strategy for the reconstruction of large novel SVs could lead to a low detection rate of smaller SV events. In this particular example, however, our optimal combination would also guarantee a high recovery rate of SNPs and small indels in the genome, according to the results of an individual genome re-sequencing project described in [4], where ∼7.4× medium reads were used to detect 3.3 million SNPs and 0.22 million indels. That is, if we focus on the optimal output of large novel SV reconstruction when designing a mixed sequencing strategy, this strategy will give us satisfying result in SNP and indel detection as well. It is also worth noting that the long reads are statistically still useful in these simulations. In general, the long reads are useful in two ways: 1) Long split-reads spanning the insertion boundary have a better chance of being correctly mapped back to the reference, thus detecting the insertion. 2) Long spanning-reads will be especially useful during novel insertion reconstruction when they cover highly repetitive regions that are longer than single medium/short reads. Fig. S1 shows some typical worst-case simulation results with and without low-coverage long reads using a same total budget. In these examples, mis-assembly around highly repetitive regions is more likely to take place without the long reads.

#### Case study: Large novel insertion reconstruction with shotgun and paired-end reads

**Figure 4:**
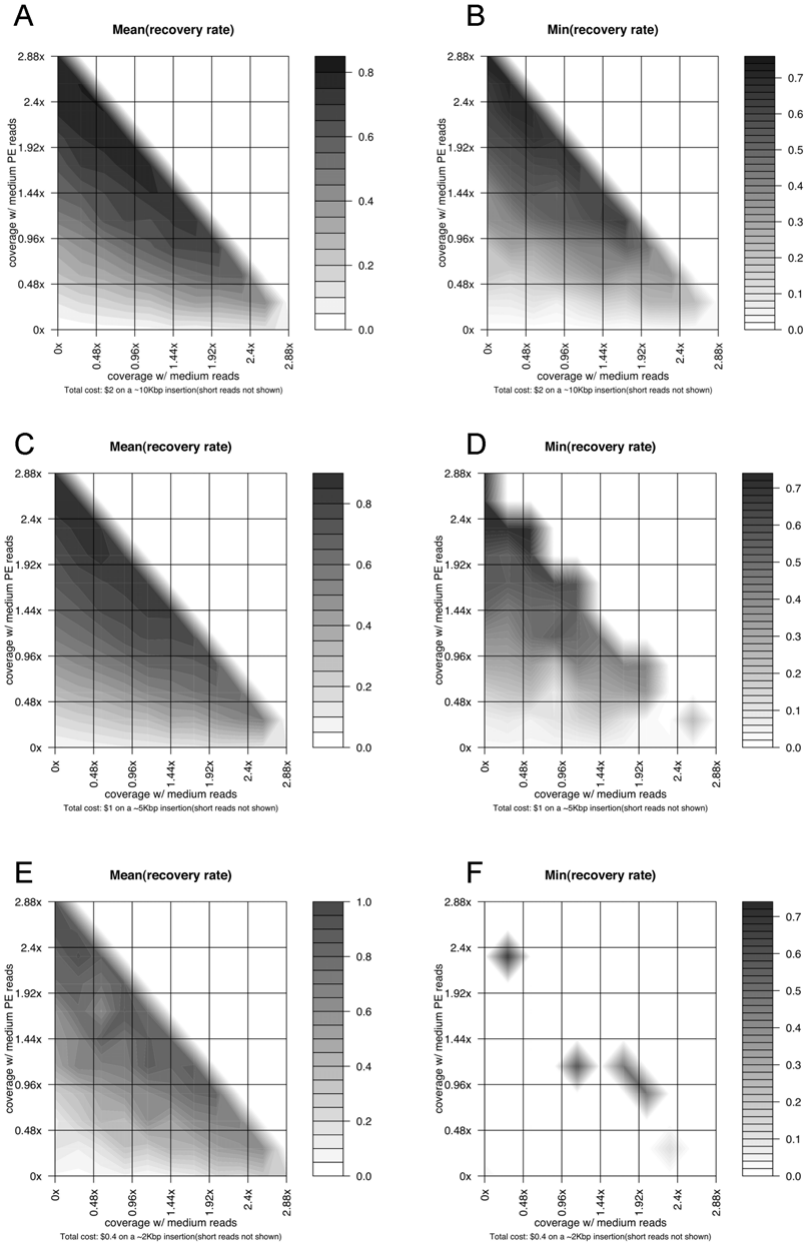
Simulation results on the reconstruction of large novel insertions using paired-end reads. (A) The same type of figure as Fig. 3B on a ∼10 Kbp novel insertion, with two axes representing the coverage of single medium and paired-end medium reads. The coverage of short reads is not explicitly shown and changes with the values of the two other two, forming a same fixed total cost. (B) The same type of figure as Fig. 4A on a ∼10 Kbp novel insertion, showing the worst-case recovery rates on the insertion region with a fixed total sequencing cost. (C) The same type of figure as Fig. 4A on a ∼5 Kbp novel insertion. (D) The same type of figure as Fig. 4B on a ∼5 Kbp novel insertion. (E) The same type of figure as Fig. 4A on a ∼2 Kbp novel insertion. (F) The same type of figure as Fig. 4B on a ∼2 Kbp novel insertion.

Similarly to Fig. 3, Fig. 4 shows the simulation results on the same insertion as well as a ∼5 Kb and a ∼2 Kb novel insertion using a combination of single and paired-end reads (medium paired-end reads with 3 Kb inserts) with a total budget of ∼$600 K (corresponding to ∼$2 on the 10 Kb novel insertion, ∼$1 on the 5 Kb insertion, and ∼$0.4 on the 2 Kb insertion). The optimal performance in reconstruction the ∼10 Kb insertion, in this case, is achieved when medium paired-end reads have ∼2.4× coverage, medium reads have ∼0.24× coverage, and short read have ∼2.4× coverage, with an average reconstruction rate of ∼0.8, which is significantly better than the results using the single reads only with the same total budget. The reconstructions on the ∼5 Kb and ∼2 Kb insertions also reach their optimal performance with a similar configuration, although their overall mean and worst-case performance differ from each other, due to the different sizes and sequence characteristics of these large novel insertions.

#### Case study: Large novel insertion reconstruction with paired-end reads using different insert sizes

**Figure 5:**
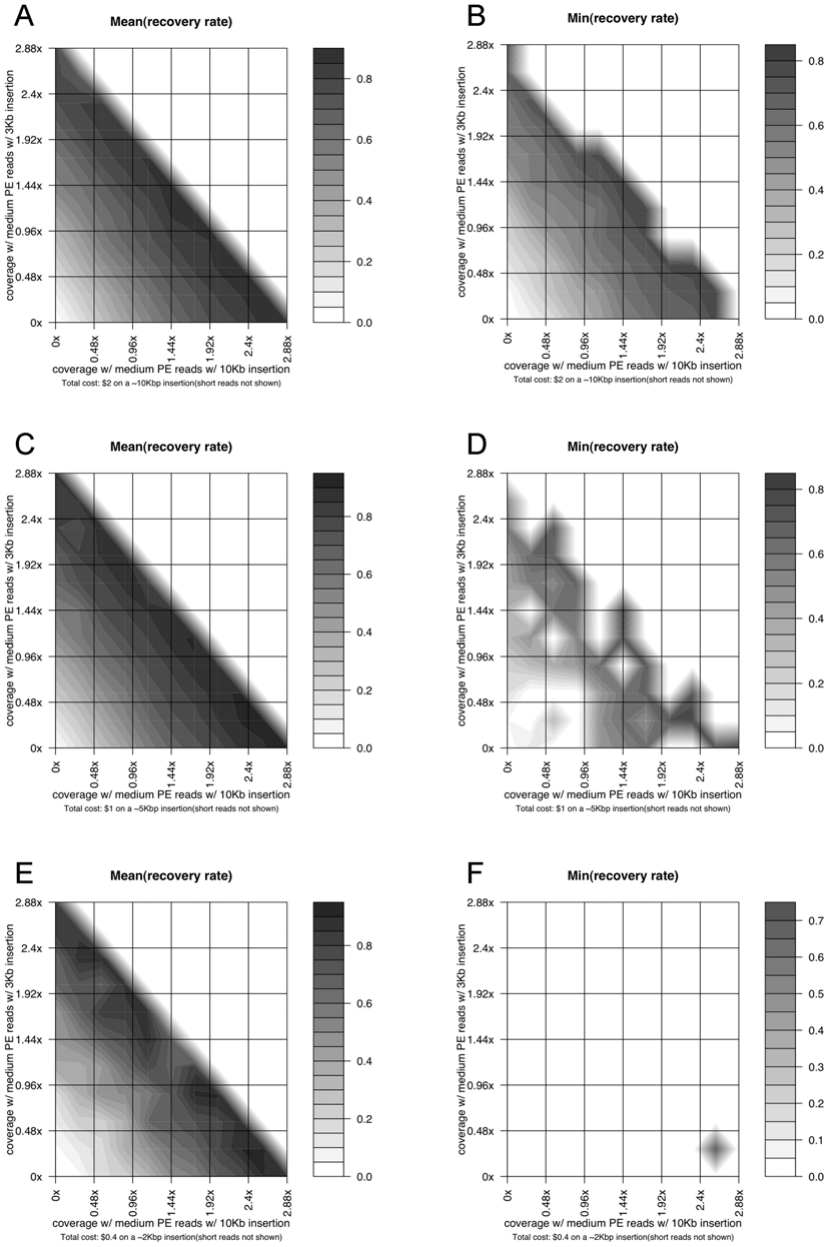
Simulation results on the reconstruction of large novel insertions using paired-end reads with different insert sizes. (A) The same type of figure as Fig. 4A on a ∼10 Kbp novel insertion, with two axes representing the coverage of paired-end medium reads with ∼10 Kbp and ∼3 Kbp inserts. The coverage of paired-end short reads (with ∼150 bp insert) is not explicitly shown and changes with the values of the two other two, forming a same fixed total cost. (B) The same type of figure as Fig. 4B on a ∼10 Kbp novel insertion, showing the worst-case recovery rates on the insertion region with a fixed total sequencing cost. (C) The same type of figure as Fig. 4A on a ∼5 Kbp novel insertion. (D) The same type of figure as Fig. 4B on a ∼5 Kbp novel insertion. (E) The same type of figure as Fig. 4A on a ∼2 Kbp novel insertion. (F) The same type of figure as Fig. 4B on a ∼2 Kbp novel insertion.

We also carried out simulations on reconstructing these novel insertion regions (∼10 Kb, ∼5 Kb, ∼2 Kb) using paired-end reads with different insert sizes (10 Kb and 3 Kb inserts for medium paired-end reads, and 150b insert for short paired-end reads). Fig. 5 shows the simulation results using different combinations of these technologies. In general, the results indicate that a low sequencing coverage of medium paired-end reads (which takes up a large fraction of the total budget due to its relatively high per-base cost) with large inserts (10 Kb in this case) and a high coverage of short paired-end reads with small inserts would be optimal for the best reconstruction performance of such novel insertions.

#### Case study on CNV analysis

**Figure 6:**
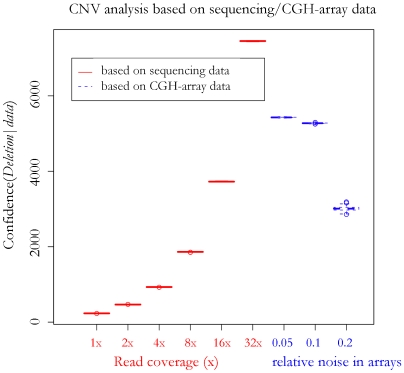
Simulation results on rearrangement and CNV analysis. Boxplot of the CNV analysis simulation results of a large (∼18 Kb) deletion in the target individual's genome. The values on the x-axis correspond to different sequencing coverage and relative noise level in the CGH arrays. The value on the y-axis indicates the confidence of using different datasets to determine that a deletion event takes place instead of a translocation event.

The second simulation focuses on the identification of genomic rearrangement events, such as deletions and translocations. CNV analysis can be used for this purpose and in this section we simulate its results based on the read-depth and signal intensity analysis of sequencing and CGH array data. Fig. 6 shows the simulation results of such analysis on a large (∼18 Kb) deletion in the target individual's genome. The analysis is based on simulated short sequence reads at different coverage, and also on simulated CGH array data with different noise levels. The log-ratio of the posterior probabilities of the deletion (as opposed to translocation) event is computed for each dataset, and used as an indicator of the confidence in determining the deletion event based on that dataset. As shown in the boxplot in Fig. 6, the confidence offered by the CGH arrays is comparable to that offered by the sequencing data with ∼16× coverage. While ∼16× coverage of short read sequencing costs ∼$0.3 M, using CGH data in this case has the advantage of achieving satisfying performance (as shown in this simulation and [3],[27] in a much more inexpensive way (∼$1000 per array).

#### Implementation and Availability

In order to be adaptive to the fast development of the experimental technologies in personal genomics, our simulation framework is modularized in such a way that it is capable of incorporating new technologies as well as adjusting the parameters for the existing ones. Also, this approach relies on the general concept of mapability data, and can be easily applied to any representative SV for similar analysis. We envision that in the future, more experimental technologies can be incorporated into this sequencing/assembly simulation and the results of such simulations can provide informative guidelines for the actual experimental design to achieve optimal assembly performance at relatively low costs. With this purpose, we have made this simulation framework downloadable at http://archive.gersteinlab.org/proj/ReSeqSim/ as a general toolbox that can be either used directly or extended easily.

## Discussion

The simulation results in the previous section are based on three sequencing technologies and an idealized array technology, and assume a specific parameterization of their characteristics and costs. Thus, the particular optimal solutions found may not be immediately applicable to a real individual genome re-sequencing project. However, these results illustrate quantitatively how we can design and run simulations to obtain guidelines for optimal experimental design in such projects.

Since our simulation approach is based on the general concept of mapability map and comparative SV reconstruction instead of on a specific organism, it can also be adapted to the comparative sequencing of a non-human genome with regard to a closely related reference. In such a study, we can first construct an artificial target genome based on estimations of its divergence from the reference, and then compute the mapability maps of those representative SVs as input to the simulation framework to find the optimal combination of technologies. Obviously, the closer the two genomes are, the more informative the simulation result would be. In cases where it is hard to estimate the divergence of the target genome from the reference, a two-step approach can be conducted: First, combined sequencing experiments will be carried out using an optimal configuration obtained from the simulation based on the “best guess”, such as another closely related genome. Second, by using the target genome constructed in the previous step, a new set of simulations can be executed and their results can guide a second round of combined sequencing which can provide a finer re-sequencing outcome when combined with the previous sequencing data. Meanwhile, our simulation framework specifically focuses on the effects of misleading reads in the SV reconstruction process, and it will be the most helpful in cases where the target and reference genome both have complex repetitive/duplicative sequence characteristics which will introduce such reads.

In this paper, we propose to optimally incorporate different experimental technologies in the design of an individual genome-sequencing project, especially for the full reconstruction of large SVs, to achieve accurate output with relatively low costs. We first describe a hybrid genome re-sequencing strategy for detecting SVs in the target genome, and then propose how we can design the optimal combination of experiments for reconstructing large SVs based on the results of semi-realistic simulations with different single and paired-end reads. We also present several examples of such simulations, focusing on the reconstruction of large novel insertions and confirmation of large deletions based on CNV analysis, which are the most challenging steps in individual re-sequencing. The simulations for actual sequencing experimental design can integrate more technologies with different characteristics, and also test the sequencing/assembly performance at different SV levels. By doing so, a set of experiments based on various technologies can be integrated to best achieve the ultimate goal of an individual genome re-sequencing project: accurately detecting all the nucleotide and structural variants in the individual's genome in a cost-efficient way. Such information will ultimately prove beneficial in understanding the genetic basis of phenotypic differences in humans.

## Materials and Methods

### The data and parameters used in the simulation

The NCBI assembly v36 [18] and the HuRef assembly [3] were used as reference and target genomes, respectively. Three sequencing technologies, long (Sanger), medium (454), and short (Illumina) sequencing, were considered with the characteristics summarized in Table 1. We also assumed that the per-base sequencing error rate increases linearly from the start to the end of a read similar to ReadSim [28], and assigned error types (insertion, deletion or substitution) randomly according to the characteristics of the sequencing technique used [6],[7],[28]. The novel SVs used in the novel insertion reconstruction simulation are ∼10 Kb, ∼5 Kb and ∼2 Kb insertion sequences in the HuRef genome [3] with variant IDs 1104685256488, 1104685222085 and 1104685613186, respectively. The deletion used in the CNV analysis simulation is a ∼18 Kb sequence in the HuRef genome with variant ID 1104685125828.

### The simulation of the sequencing/assembly of large novel insertions

Since we would be testing thousands of possible combinations of the long, medium and short sequencing technologies, it would be unrealistic (both time and space consuming) to generate for each combination all the reads from the whole target genome and then apply any existing assembler to these reads. We decided to semi-realistically simulate the assembly process of large novel insertions to achieve relatively accurate estimates in an affordable amount of time. Several difficulties need to be addressed by such a simulation: 1) One of the most time-consuming step in a real assembler is the read overlap-layout step. 2) The whole-genome sequencing experiment introduces large numbers of misleading reads that are partially similar to the reads from the targeted genomic region, which would require an huge storage space in a real assembly process.

#### The mapability data

In order to both accelerate the simulation of the overlap-layout step and simulate the whole-genome sequencing setting in a space-efficient manner, we pre-computed the mapability [29] values of all the possible sub-sequences in the reads from the inserted region. The mapability value of a sequence is the number of times this particular sequence (allowing the specified number of mismatches) appears in a genome, defined below:

**Definition 1** For a given genome *G* and a given sequence *s*, the mapability function *M(s, G, m)* is defined as the total number of occurrences of the elements in *S* in *G*, where *S = {s′|mismatch(s, s′)< = m}*. For simplicity, we also denote that *M(s, G) = M(s, G, 0)*, which is the extract occurrence of *s* in *G*.

The following lemmas are obvious:

**Lemma 1** Given a genome *G* and two sequences *s* and *s′*, if *s* contains *s′*, then *M(s, G)< = M(s′, G)*. *M(s, G) = M(s′, G)* if and only if all the occurrences of *s′* in G are within sequence *s*. An intuitive interpretation of this lemma is that if a sequence *s* contains *s′*, then *s* must occur at most the same number of times as *s′* in a genome.

**Lemma 2** Given a genome *G*, a sequence *s*, and two non-negative integers *m*, *m′*, if *m>m′*, then *M(s, G, m)> = M(s, G, m′)*. This lemma states that for any given sequence, its mapability value in a genome never decreases with an increasing mismatch threshold.

**Definition 2** For a given genome *G* and a given sequence *s*, the *k*-mapability map *MM(s, G, k, m)* of *s* with respect to *G* is a vector sequentially containing the mapability values of all the *k*-mers in *s* with a tolerance of *m* mismatches: *MM(s, G, k, m) = [M(sub(s, 0, k), G, m), M(sub(s, 1, k+1), G, m), …]*, where *sub(s, a, b)* returns the sub-sequence of s from a to b-1 (0-based index). For simplicity, we denote that *MM(s, G, k) = MM(s, G, k, 0)*, which counts exact occurrence only.

According to the above definition, *MM(s, G, k, m)* can be viewed as a set of mapability values of all the length-*k* sub-sequences in *s* allowing no more than *m* mismatches.

#### Generation of the split-/spanning-reads and computation of the mapability maps

**Figure 7:**
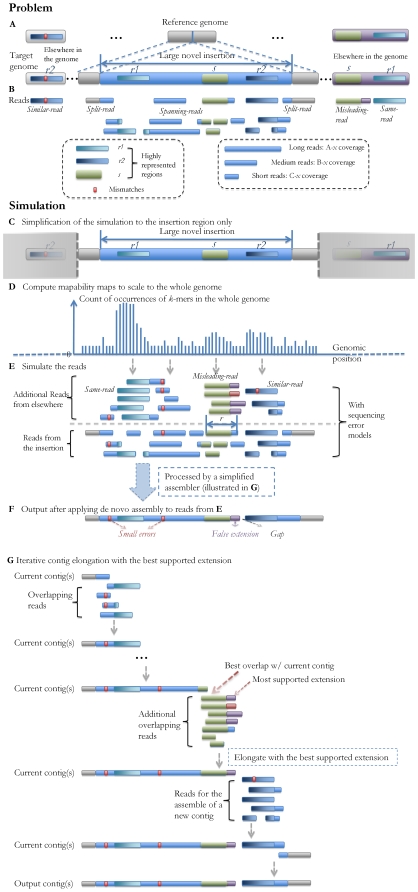
The simulation of novel insertion reconstruction. (A) A target genome with a large novel insertion. Regions *r1*, *r2*, and *s* are highly represented regions in the genome. The genomic fragments on both sides represent the existence of these regions at other locations of the genome. (B) The reads generated by whole-genome sequencing that will be included in the *de novo* assembly process of the novel insertion: the split-reads that cross the insertion boundaries, the spanning-reads from inside the insertion, the same/similar-reads from regions such as *r1* and *r2*, and misleading-reads that have the same prefix sequence *s*. (C) In the simulation, the split/spanning-reads are generated randomly from the insertion according to the coverage setting. Other locations of the target genome are not explicitly considered. (D) Mapability maps are computed for the insertion region to accelerate the future simulation steps. (E) The same/similar/misleading-reads from elsewhere in the genome are generated according to the pre-computed mapability maps. (F) The possible output contigs, which contain small sequencing errors, a false extension, and a gap. (G) A simplified assembler module to assemble all the generated reads, which extends a contig by the best overlapping reads with the most supported extension.

First, all the reads from the target insertion region are generated (Fig. 7E) based on the same setting of the long, medium and short sequencing coverages as in the problem being simulated (Fig. 7A and 7B). Second, as shown in Fig. 7D, in order to take into account the effects of the same/similar/misleading-reads from elsewhere in the genome in a whole-genome sequencing experiment, we computed the mapability maps *MM(s, G, k, m)* of the insertion region *s* (the ∼10 Kb insertion sequence with its 1 Kb up/down-stream flanking sequences), where *G* = NCBI reference genome, HuRef target genome; *k* = 25, 26, …, 800; m = 0, 2. For computational efficiency, the “mismatch” function is currently implemented to take into account only the nucleotide mismatches of two sequences with the same length. On one hand, it would be more realistic to include indel mismatches as well to represent such sequencing errors. On the other hand, we would expect that in practice most of such sequencing errors will be corrected in a preprocessing step [23].

The generated reads that align to the same genomic starting locations are grouped together and the per-position error statistics are computed, resulting in a set of read-groups that starts from different locations with their position-specific error statistics computed. These read-groups are then further combined in the de novo reconstruction process describe below.

#### Simulation of same/similar/misleading-reads in de novo reconstruction

Additional reads (same, similar and misleading) are introduced (Fig. 7E) to simulate the effects of the whole genome sequencing in Fig. 7A and 7B. The reads originating from the insertion region and the additional reads are then combined into contigs based on a heuristic read extension algorithm. This is a partial simulation of the overlap-layout-consensus/read-extension/unipath-finding step in the *de novo* assembly process [8],[10],[19],[30], where the current contig is extended based on the information of the reads that overlap with its end. The extension is only performed when there is either an unambiguous extension supported by all the overlapping reads, or when there is a sufficiently large set of reads with the longest overlap that supports the same extension.

In order to simulate such a process in a whole-genome sequencing setting, the mapability data are again utilized, as illustrated in Fig. 7D and 7E. For a highly represented region *r* in the insertion, its corresponding same/similar reads from elsewhere in the target genome are generated based on the pre-computed *M(r, TargetG, 0)* and *M(r, TargetG, 2)−M(r, TargetG, 0)* (the maximal allowed mismatch of 2 corresponds to ∼6% or lower difference between two short reads). The number of such reads are randomly generated based on the mapability values of *r*, the sequencing coverage, and the distribution *P(n, r)* of the number of reads (*n*) exactly covering a region with the same size of *r*, which can be either empirically generated based on the previously simulated reads from the inserted region, or constructed based on a theoretical Poisson distribution representing a uniform sequencing process.

The misleading-reads are generated in the following way: for a contig *c* and a read *r* that overlaps it, denote the overlapping sequence with *s*, then according to lemma 1, the unambiguous extension of *c* based on read *r* is guaranteed if and only if *M(s, TargetG) = M(r, TargetG)*, which means that the sequence *s* is always within sequence *r* in the target genome. When *M(s, TargetG)>M(r, TargetG)*, we introduce the misleading reads based on *M(s, TargetG)−M(r, TargetG)* and *P(n, r)*.

For computational efficiency, we also developed a simplified assembler module to assemble all the generated reads. As illustrated in Fig. 7G, This assembler estimates the overlaps between different reads based on their locations and the corresponding mapability values. It extends a contig by the best overlapping reads with the most supported extension, and simulates the effect of the misleading-reads in the following way: If *r* is from a paired-end read whose other end *r′* satisfies *M(r′, RefG) = 1*, we assign high confidence to *r* and always extend with its sequence. Otherwise, if the estimated number of misleading reads are significantly lower than the number reads supporting read *r* (e.g. by 2-fold), the correct extension is selected. Otherwise, if the misleading reads are over-represented, the misleading extension is chosen and the extended sequence will be different from what is in the actual inserted sequence. The longest common extension supported by all the reads is appended to *c* if neither type of reads significantly out-numbers the other. The sequencing error statistics at each position are updated accordingly in this procedure.

#### Computing the reconstruction rate of the combined result

The de novo extensions are performed by the simplified assembler described above from both ends of the insertion region, and the combined results are then compared to the actual insertion to obtain the reconstruction rate of the target region, based on the metric described in the Results section. The flanking sequences are taken into account to measure the accuracy of boundary detection. If the de novo reconstruction result does not cover the insertion boundaries, the reconstructed sequence cannot be localized in the reference genome and the reconstruction rate is set to 0. Fig. 7F shows example output contigs, which contain small sequencing errors, a false extension error due to the misleading-reads introduced by an highly represented region inside the insertion, and a gap due to both the false extension and the low-coverage of sequencing in that particular region.

### The simulation of CNV analysis

In this simulation, we assume that the boundaries of a large deletion event have already been identified by sequence reads, and we are simulating the process of determining whether this is a deletion or translocation event, based on the short reads alone or on the idealized CGH data. The reads are generated in a similar fashion as described in the previous section, without considering sequencing errors for simplicity. The idealized CGH signal of a corresponding region *r* is defined as Gaussian variable with mean *M(r, TargetG)*, and noise/standard deviation = 0.05, 0.1, 0.2. For each dataset, the log-ratio of the posterior probability of the deletion event is computed to represent the confidence level provided by each dataset for determining that deletion. These confidence levels are computed according the following formulas:
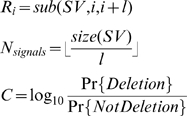


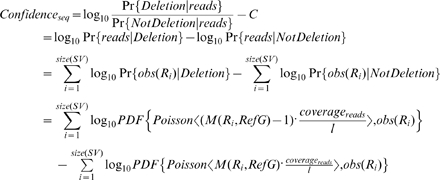


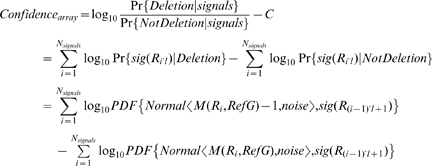
where *sub(s, a, b)* returns the sub-sequence of s from a to b-1 (1-based index), *l* is the length of the short read, *SV* stands for the deleted region, *coverage_reads_* is the sequencing coverage, *obs(r)* is the number of observed reads that are the same as *r*, *sig(r)* is the normalized CGH-array signal of probe *r*, *PDF{D, v}* is the probability density/mass function of the distribution *D* at value *v*, and *RefG/TargetG* refers to the reference/target genome.

## Supporting Information

Figure S1 MM values and worst case reconstruction examples of a 10 Kb novel insertion.
(0.08 MB PDF)
