# Supplementary material for: Integrating Sequencing Technologies in Personal Genomics: Optimal Low Cost Reconstruction of Structural Variants
Source: PLoS Comput Biol. 2009 Jul 10;5(7):e1000432. doi: 10.1371/journal.pcbi.1000432 (PMC2700963; doi:10.1371/journal.pcbi.1000432)
Supplement: Figure S1 — MM values and worst case reconstruction examples of a 10 Kb novel insertion. (0.08 MB PDF) [file pcbi.1000432.s001.pdf]

Integrating sequencing technologies in personal genomics: optimal low cost reconstruction of structural variants

Jiang Du Robert D. Bjornson Zhengdong D. Zhang Yong Kong
Michael Snyder Mark B. Gerstein

Figure S1

MM values and worst case reconstruction examples of a 10Kb novel insertion.
(A) Mapability values for all the 30mers of a $\sim$ 10Kb novel insertion (Variant ID in Huref: 1104685256488, with 1000 flanking sequences): $MM(flanking_{1000bp}(Ins), G_{hg18}, 30, 0)$. The insertion region is shown in blue.
(B) and (C) show the simulation results in reconstructing this region with a same total budget of ~$7. The solid blue lines are the assembled contigs that can be localized back to this insertion, with solid red lines for the parts that do not match due to mis-assembly. The dotted blue lines are the contigs that cannot be localized back to this insertion, with the dotted red lines representing the parts that do not match.
(B) Typical worst-case reconstruction result with $\sim$ 0x long reads, $\sim$ 7x medium reads, and $\sim$ 17.5x short reads.
(C) Typical worst-case reconstruction result with $\sim$ 0.05x long reads, $\sim$ 7x medium reads, and $\sim$ 10x short reads.

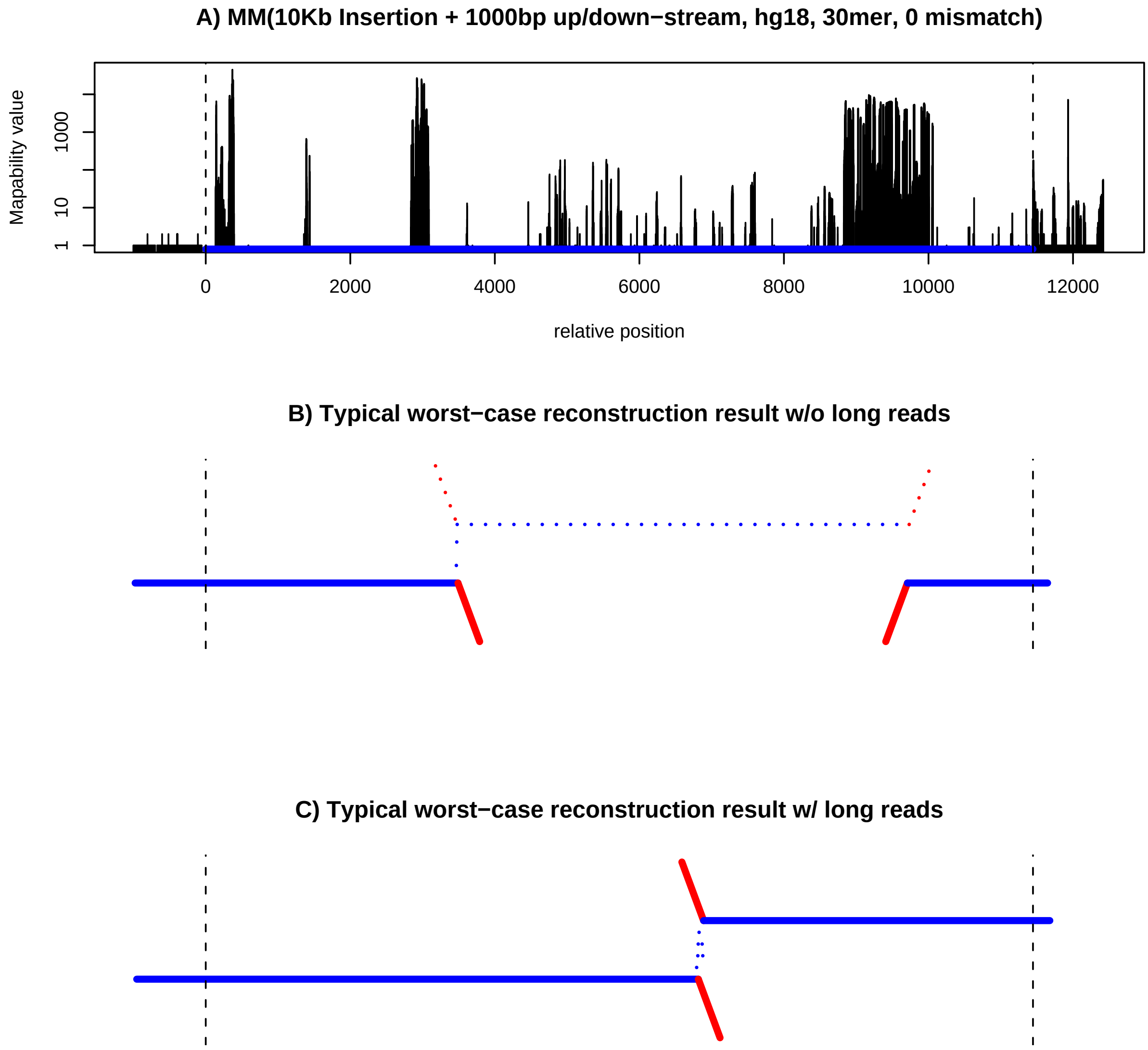

Figure S1: MM values and worst case reconstruction examples of a 10Kb novel insertion.
